# How do antimalarial drugs reach their intracellular targets?

**DOI:** 10.3389/fphar.2015.00091

**Published:** 2015-05-05

**Authors:** Katherine Basore, Yang Cheng, Ambuj K. Kushwaha, Son T. Nguyen, Sanjay A. Desai

**Affiliations:** ^1^The Laboratory of Malaria and Vector Research, National Institute of Allergy and Infectious Diseases, National Institutes of Health, Rockville, MD, USA; ^2^Microbiotix Inc., Worcester, MA, USA

**Keywords:** antimalarials, drug uptake, plasmodial surface anion channel, plasmodium falciparum, drug absorption, lipid diffusion of drugs

## Abstract

Drugs represent the primary treatment available for human malaria, as caused by *Plasmodium* spp. Currently approved drugs and antimalarial drug leads generally work against parasite enzymes or activities within infected erythrocytes. To reach their specific targets, these chemicals must cross at least three membranes beginning with the host cell membrane. Uptake at each membrane may involve partitioning and diffusion through the lipid bilayer or facilitated transport through channels or carriers. Here, we review the features of available antimalarials and examine whether transporters may be required for their uptake. Our computational analysis suggests that most antimalarials have high intrinsic membrane permeability, obviating the need for uptake via transporters; a subset of compounds appear to require facilitated uptake. We also review parasite and host transporters that may contribute to drug uptake. Broad permeability channels at the erythrocyte and parasitophorous vacuolar membranes of infected cells relax permeability constraints on antimalarial drug design; however, this uptake mechanism is prone to acquired resistance as the parasite may alter channel activity to reduce drug uptake. A better understanding of how antimalarial drugs reach their intracellular targets is critical to prioritizing drug leads for antimalarial development and may reveal new targets for therapeutic intervention.

## Introduction

Malaria remains a leading infectious cause of morbidity and mortality worldwide. Because an effective vaccine is not available, treatment with drugs or drug combinations remains the mainstay of malaria control (Visser et al., 2014). With primaquine as a notable exception (Vale et al., 2009), the available drugs work primarily against bloodstream parasite stages, which invade and replicate within human erythrocytes. Development within erythrocytes protects the pathogen from host immune responses (Hafalla et al., 2011), but limits parasite access to nutrients and other solutes in plasma (Homewood and Neame, 1974; Kutner et al., 1982; Desai et al., 2000; Staines et al., 2007). Antimalarial drugs have focused on these bloodstream parasites because these stages are responsible for nearly all the clinical sequelae of malaria. To be effective, the drug must reach appropriate plasma levels, enter the infected cell and access its intracellular target, inhibit one or more essential parasite activities selectively, and produce rapid killing. To be useful in the developing world and to impact global health (Burrows et al., 2013), there are a number of additional constraints on antimalarial drugs: (1) they must be affordable, ideally costing less than $0.25 USD for a single treatment course, (2) the drug or drug combination should cure infection without leading to dormant forms that yield recurrent illness or “recrudescence” (White, 2008), (3) the risk of acquired parasite resistance must be low, (4) the drug should be safe and nontoxic in children and pregnant women, groups prone to more severe malaria, (5) the drug should be effective against multiple parasite species, ideally preventing relapses associated with *P. vivax* and *P. ovale*, (6) the drug should be effective in both acute therapy and prophylaxis, as needed by travelers to endemic areas, (7) the formulation should be stable and distributed easily without refrigeration or other precautions, (8) oral administration of ideally a single curative dose is strongly preferred, and (9) pharmacokinetic properties such as half-life and route of excretion should be optimized to ensure cure, but avoid development of parasite resistance. This long list of desired attributes has thwarted drug discovery and development in malaria, accounting for the limited number of approved drugs available for treatment of this important disease.

In light of these major hurdles, the precise mechanism of action, access to the parasite target, and possible resistance mechanisms are often not explored as extensively as might otherwise be desired. Here, we consider the fundamental question of how drugs reach their parasite target. Nearly all antimalarials must cross multiple membrane barriers to reach their parasite target within infected erythrocytes. We report that most approved drugs and drug leads have chemical properties that allow free diffusion through lipid bilayers to reach their intracellular targets; transporters at specific membranes also contribute to uptake of more hydrophilic agents.

## Two Mechanisms of Drug Uptake by Cells

Small organic solutes such as nutrients and drugs enter cells by crossing the surface membrane, which is made of phospholipids, cholesterol, and other amphipathic molecules arranged in a ∼50 Å bilayer. Biological membranes also contain embedded proteins that may function as receptors and solute transporters. Thus, nutrients and drugs may cross the membrane either by diffusion through the lipid bilayer portion or by use of a carrier or transporter protein, a process commonly referred to as facilitated diffusion or active transport if external energy is used in the transport cycle. Historically, lipid-based diffusion was thought to be the main route for uptake of drugs, but with the identification of organic solute carriers (Hediger et al., 2004; Dobson and Kell, 2008), facilitated diffusion has become increasingly appreciated. Certainly, these mechanisms may coexist, depending on the cell type and the specific drug in question (Sugano et al., 2010).

A few characteristics of these two mechanisms may help determine which route is dominant for a given drug. Lipid-based diffusion is generally restricted to uncharged chemicals or drugs because charged solutes do not partition easily into the lipid core of a membrane bilayer. Because specific binding sites are not involved, lipid-based diffusion is generally not saturable, not subject to inhibition, and not sensitive to drug stereospecificity. Although there are cell-specific differences in lipid composition and bilayer structure, the physicochemical properties of biological membranes are largely conserved; as a result, lipid-based diffusion of a drug generally does not vary between cell types. In contrast, carrier-mediated uptake is typically saturable, amenable to pharmacological inhibition, and affected by drug stereospecificity. Because expression of solute carriers is specific to certain cell types and differs amongst higher organisms, drug uptake by this mechanism may vary dramatically from cell to cell. Both mechanisms allow movement of a drug by mass action, i.e., down its concentration gradient, but some carriers can transport solutes against their concentration gradient by coupling movement to ATP hydrolysis or the flux of another solute.

While carrier-mediated uptake cannot be readily predicted by knowledge of the physical and chemical properties of a drug, significant effort has been invested in predicting lipid-based diffusion of drugs. Because lipid diffusion correlates with passive intestinal absorption, ensuring adequate permeability by this mechanism is particularly important during the drug discovery and development process. Compounds with high lipid diffusion are more likely to be orally absorbed and to reach therapeutic levels at their target site. One approach has been to use *in vitro* assays such as the Caco-2 intestinal cell permeability assay and the parallel artificial membrane permeability assay, PAMPA (Kansy et al., 2004; van Breemen and Li, 2005). Since their introduction, these assays have helped reduce drug development program failures by revealing poor pharmacokinetic properties at an early stage. Nevertheless, these assays exhibit significant lab-to-lab variability and remain imperfect predictors of oral absorption in animals.

Another important approach to predicting lipid diffusion has involved computational methods that consider the physicochemical properties of drug molecules. One widely recognized example is the Lipinski’s “Rule of 5,” which examined a large collection of late stage drug candidates with established oral bioavailability to identify simple parameters that predict passive intestinal absorption (Lipinski et al., 2001). They defined threshold values of four parameters—calculated LogP (CLogP), molecular weight, number of H-bond donors, and number of H-bond acceptors—that predicted good oral absorption. A limitation of this approach is that it does not quantify the degree of lipid permeability of well-absorbed compounds; it also leaves many compounds in an uncertain category as they satisfy some but not all of the criteria. Thus, a number of increasingly complex algorithms have been developed (Camenisch et al., 1998; Zhao et al., 2002; Yan et al., 2008). An important problem with these algorithms is that the parameters used—polar surface area (PSA), polarizability, various measures of lipophilicity (logP, log *D*, non-PSA, etc.), molecular volume, and other parameters used in the Lipinski Rule of 5—are interrelated, so the relative importance of each parameter is unclear.

Another problem is that calculation or measurement of some parameters is not readily available to all workers, especially those in academia. Commercial software is available to calculate these parameters and estimates oral bioavailability, distribution in mammalian organs, metabolism, and excretion; however, such software is expensive and typically available only to workers in the pharmaceutical sector.

## Predicted Lipid Permeability of Antimalarials

**FIGURE 1:**
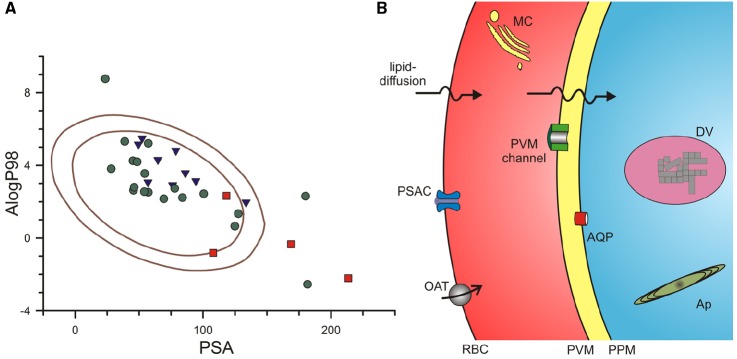
**Routes used by antimalarial compounds to reach their intracellular parasite targets. (A)** Plot of PSA vs. AlogP98 for antimalarial compounds, calculated using Accelrys Draw 4.2. Solid lines represent the 95 and 99% confidence ellipses for drugs with good absorption (inner and outer ellipses, respectively), as determined by (Egan et al., 2000). Notice that approved antimalarial drugs generally fall within these ellipses (green circles), indicating a high likelihood for adequate lipid-diffusion to reach intracellular targets. Compounds in the medicines for malaria (MMV) pipeline and key antimalarial toxins are shown as blue triangles and red squares, respectively. Approved drugs shown: chloroquine, mefloquine, quinine, artemisinin, dihydroartemisinin, artesunate, artemether, atovaquone, proguanil, naphthoquine, amodiaquine, piperaquine, doxycycline**, clindamycin, azithromycin**, sulfadoxine, lumefantrine^**^, pyrimethamine, pyronaridine. MMV pipeline compounds: DSM265, MMV390048, NITD609, KAF156, P218^*^, SJ733, PA21A092, OZ439, tafenoquine. Antimalarial toxins: fosmidomycin^*^, blasticidin S^**^, leupeptin^**^, pentamidine. Single asterisk indicates compound is outside the 95% confidence ellipse only; Double asterisk indicates compound is outside both ellipses. **(B)** Schematic showing membrane barriers and transporters available for drug uptake at key membranes of the infected erythrocyte. Drugs may enter via lipid-diffusion (upper arrows) or through PSAC or a host transporter (e.g., organic anion transporter, OAT) at the erythrocyte membrane, the PVM channel at the parasitophorous vacuolar membrane (PVM), and one or more transporters (e.g., aquaglyceroporin, AQP) at the parasite plasma membrane (PPM). Drugs may act within parasite organelles such as the digestive vacuole (DV, shown containing crystalline hemozoin) or the multilamellar apicoplast (Ap), imposing additional membranous barriers; drug transporters are also present at these membranes (not shown). A parasite-generated Maurer’s cleft (MC) is shown in the host cytosol. Transporters for nutrients such as sugars, amino acids, and nucleobases are also present at some membranes (not shown); certain drugs may use these nutrient transporters for uptake.

To explore whether existing antimalarials use lipid-based or carrier-mediated uptake to reach their intracellular targets, we used a computational approach that overcomes some of these limitations. The multivariate approach developed by Egan et al. (2000) identified AlogP98, a computationally simpler method of estimating logP values (Ghose et al., 1998), and PSA as two independent parameters that contribute to lipid-based diffusion (Egan et al., 2000). They then defined ranges of these values that correlated with absorption by examining collections of well-absorbed and poorly-absorbed drugs. Well-absorbed drugs reproducibly exhibited values for these parameters that fell within an ellipse in a two-dimensional plot; Figure 1A shows the 95 and 99% confidence ellipses for well-absorbed drugs taken from their analyses (areas encoded by the inner and outer solid curves, respectively). Compounds from their poorly-absorbed collection were consistently outside of these ellipses, with only 1 compound lying just within their 95% ellipse.

We then calculated AlogP98 and PSA for approved antimalarial drugs, drug leads and compounds in the Medicines for Malaria pipeline (Burrows et al., 2013), and other antimalarial agents of interest. These values were added to Figure 1A, using distinct symbols for each class of compounds. Notably, most of the compounds fall within the good absorption 95% confidence ellipse, a finding consistent with the need to cross multiple membranes to reach their intracellular parasite drug targets (Figure 1B, discussed in detail below). Without good absorption at these multiple membranes, a compound is not likely to interfere with parasite propagation under either *in vitro* or *in vivo* conditions.

Figure 1A shows that three approved antimalarials and 4 drug leads are predicted to have marginal or poor absorption (symbols outside of the inner ellipse). How do these compounds reach their intracellular targets to produce parasite killing? As discussed below, three of these compounds, blasticidin S, leupeptin, and fosmidomycin, cross the host erythrocyte membrane through a parasite-induced channel (Hill et al., 2007; Lisk et al., 2008, 2010; Baumeister et al., 2011; Nair et al., 2011). Three approved antimalarial drugs, doxycycline, azithromycin, and lumefantrine, are predicted to be poorly absorbed (filled circles outside outer ellipse, Figure 1A), but their uptake by infected cells has not been directly studied; each has known problems with oral absorption, with facilitated or active transport already established for some cell types (Meshali and Attia, 1978; Farmer et al., 1992; Ezzet et al., 2000; Wahajuddin et al., 2014). Finally, the preclinical antimalarial candidate P218 falls just outside the inner ellipse; because it is reported to have good oral bioavailability (Yuthavong et al., 2012), this compound may also use facilitated transport on carriers or channels.

## At Least Three Membranes to be Crossed

Antimalarials with targets within the intracellular parasite must sequentially traverse three membranes by lipid diffusion, flux through one or more transporters, or both mechanisms in parallel (Figure 1B). The solute transport properties of these membranes are reviewed next.

### The Host Erythrocyte Membrane

In addition to organic solute carriers endogenous to human erythrocytes (Figure 1B, OAT; Heijn et al., 1992; Saxena and Henderson, 1996; Zipprer et al., 2014), an unusual ion and nutrient channel known as the plasmodial surface anion channel (PSAC) is induced on the host membrane of infected cells to increase ion and organic solute permeabilities (Overman, 1948; Ginsburg et al., 1983; Desai et al., 2000). PSAC activity has recently been linked to the parasite *clag* gene family (Nguitragool et al., 2011). Solutes transported by this channel may be charged or uncharged and may have molecular weights over 600 dal (Cohn et al., 2003), suggesting that this route may be accessible to many drugs. Remarkably, the small Na^+^ ion is excluded by poorly understood mechanisms, though uptake studies at basic pH values suggest a contribution of electrostatic repulsion (Cohn et al., 2003). Although the channel functions primarily in uptake of essential nutrients (Pillai et al., 2012, 2013), a number of drugs or drug leads also have demonstrated uptake via this channel (Stead et al., 2001; Biagini et al., 2003; Hill et al., 2007; Lisk et al., 2008, 2010; Baumeister et al., 2011; Dana et al., 2014). Blasticidin S and leupeptin, two agents that require PSAC-mediated uptake, have been used to select transport mutants and provide molecular insights into solute transport by this channel (Mira-Martinez et al., 2013; Sharma et al., 2013). These transport mutants preserve uptake of essential nutrients, but produce resistance by reducing uptake of the toxin selectively. They also support an essential role of PSAC because complete loss-of-function mutants have not been generated *in vitro* or identified in *ex vivo* samples (Desai, 2012).

Plasmodial surface anion channel or other parasite-associated channels may contribute to uptake of existing antimalarial drugs such as artemisinin (Vyas et al., 2002; Duranton et al., 2005). With evolving guidelines for the design of antimalarials with greater water solubility (Burrows et al., 2013), future drugs may depend on PSAC-mediated uptake to a greater extent. An important concern is whether resistance and clinical failures may arise quickly through the selection of PSAC mutants. The availability of potent and specific PSAC inhibitors permits easy determination of whether a drug requires channel-mediated uptake (Pillai et al., 2010), using either radiolabeled drug uptake or *in vitro* growth inhibition studies in combination experiments with PSAC inhibitors (Lisk et al., 2010). Given the risk of acquired drug resistance through reduced uptake, drug discovery programs should routinely test their lead compounds for uptake via this channel.

### The Parasitophorous Vacuolar Membrane

Drugs that have entered the erythrocyte cytosol then encounter the parasitophorous vacuolar membrane (PVM), which surrounds the parasite throughout its intracellular cycle. Because the PVM is closely apposed to the underlying parasite plasma membrane (Figure 1B), its transport properties have been relatively difficult to characterize. Patch-clamp studies have however, revealed a single large conductance ion channel at the PVM (Desai et al., 1993). Present at high density on this membrane, this channel mediates the high capacity transport of both cations and anions; inorganic and organic ions including tris^+^, lysine^+^, and glucuronate^–^ are transported with indistinguishable rates. Subsequent reconstitution into planar lipid bilayers yielded a size cut-off of 1400 dal for permeating solutes (Desai and Rosenberg, 1997). Consistent with these electrophysiological studies, biochemical measurements also suggest free exchange of large organic solutes at the PVM (Nyalwidhe et al., 2002). Although the molecular basis of this channel remains unknown, it appears to be highly conserved in apicomplexan parasites, with similar activity observed in *Toxoplasma gondii* (Schwab et al., 1994). These transport features suggest that most, if not all, antimalarial drugs will readily cross the PVM through this channel. The PVM channel does not appear to be inhibited by high concentrations of quinine, chloroquine or artemisinin, suggesting that it may also be an unexplored drug target (Desai and Rosenberg, 1997).

A recently identified plasmodium translocon of exported proteins (PTEX) is also found at the PVM (Goldberg and Cowman, 2010). PTEX functions to export many parasite proteins to the host cytosol (Beck et al., 2014; Elsworth et al., 2014), enabling a dramatic remodeling of the erythrocyte. Because protein translocons in other organisms allow ion flux (Simon and Blobel, 1991, 1992), one possibility is that PVM channel and PTEX activities are mediated by the same molecular complex.

### The Parasite Plasma Membrane

Drug transport at the third membranous barrier, the parasite plasma membrane (Figure 1B), is poorly understood. Although a number of transporters and pumps have been identified at this membrane through either biochemical studies of freed parasites or through computational approaches that search for orthologs of known solute transporters (Woodrow et al., 1999; Rager et al., 2001), most known transporters at this membrane are thought to have restricted substrate specificities and may not mediate drug uptake. A possible exception is a parasite aquaglyceroporin, PfAQP, at the parasite plasma membrane (Hansen et al., 2002). This atypical water channel transports glycerol, sugar alcohols with up to five carbons and various toxic compounds containing carbonyl groups (Pavlovic-Djuranovic et al., 2006; Zeuthen et al., 2006). A subset of antimalarial agents may therefore access parasite cytosol via this promiscuous channel, as has also been reported in *Trypanosoma brucei* (Munday et al., 2014).

Further evidence for limited permeability of the parasite plasma membrane comes from studies with fosmidomycin, a drug lead with poor lipid-based uptake (Figure 1A). This agent targets isoprenoid biosynthesis in the parasite apicoplast, but is surprisingly inactive against related parasites such as *Toxoplasma gondii* that also depend on this biosynthetic pathway. A recent study transfected *T. gondii* parasites with a bacterial glycerol-3-phosphate transporter and targeted it for the parasite plasma membrane (Nair et al., 2011). Interestingly, expression of this transporter allowed fosmidomycin uptake and conferred a >100-fold increase in drug action against that parasite. It is unclear how fosmidomycin and other poorly permeable drug leads such as blasticidin S, leupeptin, and doxycycline (Figure 1A) cross the parasite plasma membrane in malaria parasites, but drug conjugation with the permeability-promoting octaarginine peptide suggests that this membrane may be a limiting barrier in malaria parasites also (Sparr et al., 2013). These agents and other drugs may cross the parasite plasma membrane on one of several nutrient carriers that have been localized to this membrane (Woodrow et al., 1999; Carter et al., 2000)

Many drugs act against targets with parasite organelles and must therefore cross one or more additional membranes after uptake at the parasite plasma membrane. For example, a number of drugs including chloroquine and mefloquine act in the parasite digestive vacuole and must cross this organelle’s membrane to be effective. Two intensively studied transporters, PfCRT and PfMDR1 (also known as Pgh-1), localize to this membrane and carry mutations or copy number variations confering resistance to various antimalarials (Duraisingh and Cowman, 2005; Sa et al., 2009). These altered transporters may confer resistance by pumping drugs out of the vacuole, confirming the clinical relevance of drug permeability considerations.

## Conclusion

Because the targets of most antimalarial drugs and drug leads are proteins within the intracellular bloodstream parasite, these compounds must cross at least three membranes to effect parasite killing. They may cross each of these membranous barriers by either lipid-diffusion or by use of one or more transporters of host or parasite origin. Our analysis shows that most existing antimalarials have physicochemical properties consistent with lipid-diffusion at rates sufficient for oral uptake and accumulation within the infected erythrocytes. Broad selectivity ion channels at the host plasma membrane and the PVM provide a route for diverse antimalarials that might not readily enter other cells. Many existing antimalarial drugs will then also need to cross additional membranes within the parasite to reach their specific targets parasite organelles such as the digestive vacuole. Transporters and channels that facilitate drug uptake by infected cells have been identified and are under active study. Together with new approaches to estimate lipid-diffusion of drugs, a clear understanding of how drugs reach their parasite targets is now possible. This understanding will help prioritize drug leads, improve therapeutic algorithms with drug combinations, and provide critical insights into drug resistance mechanisms.

### Conflict of Interest Statement

The authors declare that the research was conducted in the absence of any commercial or financial relationships that could be construed as a potential conflict of interest.
